# Case report: rapid growth of malignant phyllodes tumor

**DOI:** 10.1093/jscr/rjaf082

**Published:** 2025-02-28

**Authors:** Niyousha Ahmadi, Joanna Sajdlowska, Pasha Shenasan, John Veltri, Nadra Moulayes

**Affiliations:** General Surgery Department, St. Joseph University Medical Center, 703 Main St, Paterson, NJ 07503, United States; General Surgery Department, St. Joseph University Medical Center, 703 Main St, Paterson, NJ 07503, United States; General Surgery Department, St. Joseph University Medical Center, 703 Main St, Paterson, NJ 07503, United States; General Surgery Department, St. Joseph University Medical Center, 703 Main St, Paterson, NJ 07503, United States; General Surgery Department, St. Joseph University Medical Center, 703 Main St, Paterson, NJ 07503, United States

**Keywords:** breast cancer, breast mass, mastectomy, phyllodes

## Abstract

Phyllodes tumors (PT) are exceedingly rare fibroepithelial neoplasms that arise from breast stromal tissue, primarily occurring in women in the fourth and fifth decade. This poses a diagnostic challenge among younger women with breast masses, such as those in their late teens to early thirties, where benign breast masses such as fibroadenomas are more common. In this report, a 19-year-old female presented with a rapidly enlarging complex mass of the right breast over the course of two months. An ultrasound revealed a mass measuring 19 × 17 × 23 cm, with multiple solid and cystic components. The mass was classified as BI-RADS 4. Due to the continuous growth, the patient underwent total right mastectomy. Final pathology revealed a malignant phyllodes tumor measuring 24 × 12 × 7 cm with cystic component. Phyllodes tumor management remains complex, requiring a tailored multidisciplinary approach based on individual tumor characteristics and patient factors.

## Introduction

Phyllodes tumors (PT) are exceedingly rare fibroepithelial neoplasms that arise from breast stromal tissue, primarily occurring in women in their fourth and fifth decade [1, 2]. Histologically, these tumors can resemble both fibroadenomas and sarcomas on the benign and malignant sides of the spectrum, respectively [3]. Consequently, both definitive diagnosis and treatment necessitate surgical excision of the mass. However, there are no established evidence-based guidelines on surgical margins, the effect of adjuvant therapy on disease recurrence, overall prognosis, or the length of follow-up recommended after tumor resection [4, 5]. Management often relies on clinician judgment and requires tailored consideration of individual tumor characteristics and patient factors. Here, we present a case of a rapidly growing malignant phyllodes tumor in a young female, characterized by necrosis and ulceration, which required mastectomy.

## Case report

**Figure 1:**
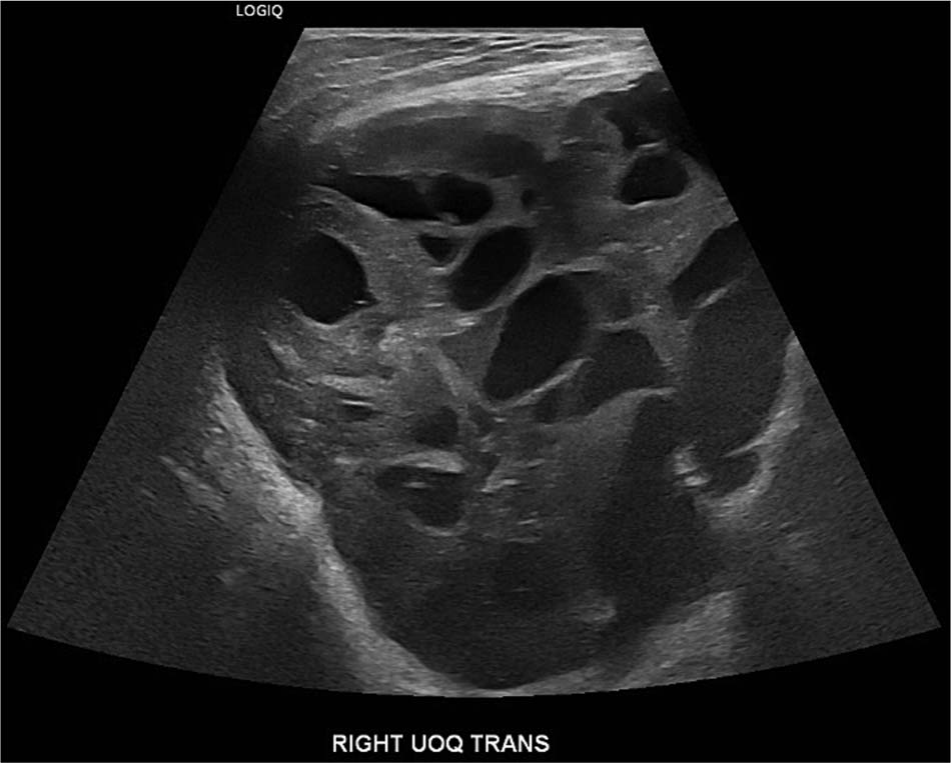
An ultrasound at that time revealed a large complex mass measuring 19 × 17 × 23 cm, with multiple solid and cystic components and no increased vascularity, consistent with BI-RADS 4.

**Figure 2:**
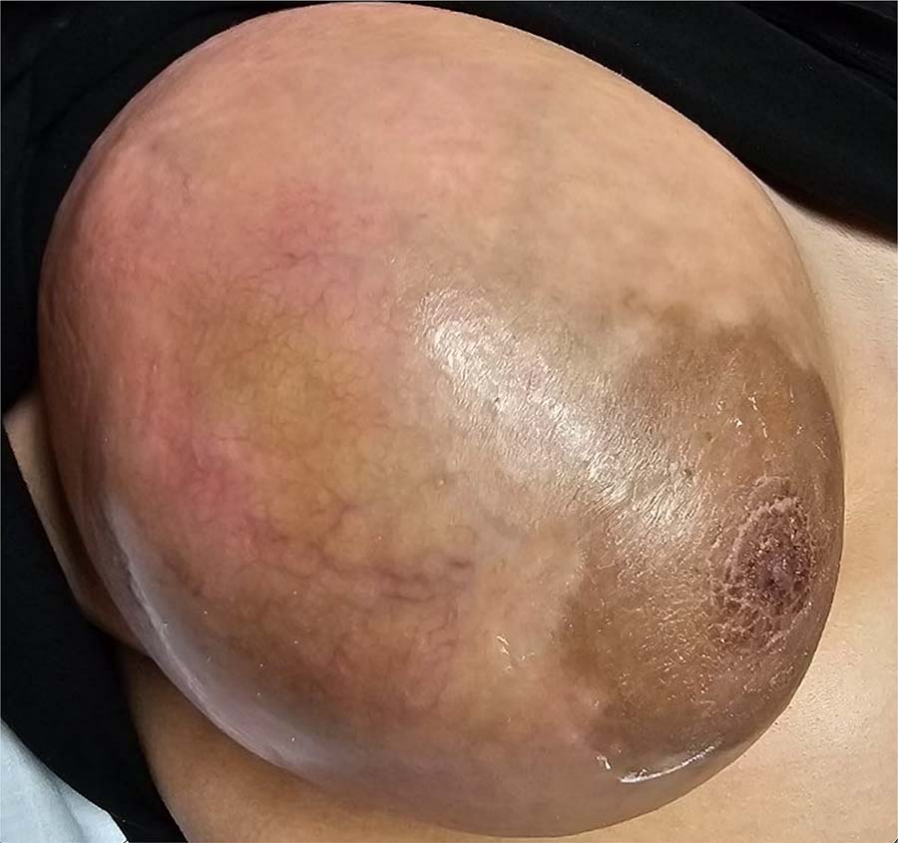
Preoperative image of breast with increased vascularity, erythema, and skin ulceration.

A 19-year-old Hispanic female with no significant past medical history presented to the emergency department with a rapidly growing right breast mass. She reported first noticing the mass at age fourteen, at which point it was small and stable in size until approximately two months prior, when it began to grow and become painful, ultimately causing complete distortion of the breast and nipple areolar complex (Fig. 1). An ultrasound at that time revealed a large complex mass measuring 19 × 17 × 23 cm, with multiple solid and cystic components and no increased vascularity (Fig. 2). The mass was classified as BI-RADS 4.

The patient was referred to the breast surgery clinic for follow-up, where a full workup was recommended, including bilateral breast and axilla ultrasound and core needle biopsy. Before the appointments, the patient returned to the emergency department due to persistent bleeding from an area of ulceration over the right breast. An ultrasound-guided biopsy of the mass was inconclusive, showing mostly fibroblastic proliferation with focally extensive fibrin and red blood cells, and fragments of fibroadipose tissue without any identifiable breast tissue.

**Figure 3:**
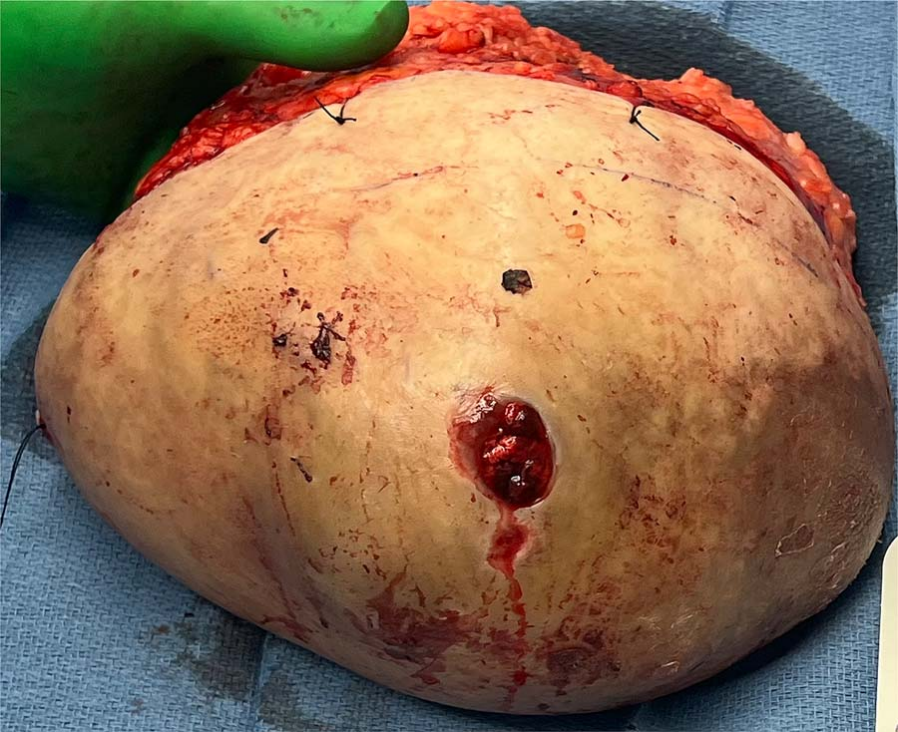
Specimen status post right mastectomy area of ulceration lateral to the nipple areolar complex, confirmed to be 24 × 12 × 7 cm with cystic component on final pathology evaluation.

Due to the continuous growth, associated skin ulceration, and bleeding, as seen in Fig. 2, the patient was taken urgently to the operating room for a total right mastectomy (Fig. 3). During intraoperative examination, multiple enlarged lymph nodes in the axilla and the lateral chest wall were found to be enlarged and excised as part of the specimen. Final pathology revealed a malignant phyllodes tumor measuring 24 × 12 × 7 cm with cystic components. The tumor was 1 mm away from the posterior margin, 8 mm away from the medial margin, 1.1 cm away from the lateral margin, 1.5 cm away from the superior margin, and 1 cm away from the inferior margin. All five lymph nodes were negative for metastases and only reactive. A metastatic workup with CT scans of the chest, abdomen, and pelvis was negative. Multidisciplinary tumor board discussions concluded that the patient did not require adjuvant therapy or further excision but would benefit from close follow-up to monitor for disease recurrence.

## Discussion

Phyllodes tumors of the breast, also known as cystosarcoma phyllodes, are rare fibroepithelial neoplasms of stromal tissue in the breast, comprising 0.3%–1% of female breast tumors [1]. They are classified as benign, borderline, or malignant and share histological features with breast fibroadenomas on the benign end of the spectrum and sarcomas on the malignant end.

The peak incidence of phyllodes tumors is in middle-aged women between 40 and 50 years old, and they are rarely found in the elderly or adolescents [2]. This poses an additional diagnostic challenge when evaluating breast masses in younger women, such as those in their late teens to early 30s, where benign breast masses such as fibroadenomas are more common.

Both fibroadenomas and phyllodes tumors present as well-circumscribed masses in the breast and can be difficult to distinguish on mammography. A history of rapid growth, larger-sized masses, and sonographic features such as a lobulated shape, a heterogeneous internal echo pattern, and the absence of microcalcifications can support a diagnosis of phyllodes tumor over fibroadenoma [3]. Core needle biopsy is also often used in the initial diagnostic workup; however, as in our patient’s case, definitive diagnosis relies on histopathological examination. Microscopically, phyllodes tumors are characterized by stromal overgrowth and a leaf-like architecture. Malignant phyllodes tumors are distinguished by high stromal cellularity, atypical stromal cells, and significant mitotic activity [1].

Current management practices for phyllodes tumors show a lack of consensus and evidence-based guidelines regarding appropriate surgical margins to ensure complete excision and minimize recurrence risk [4, 5]. Historically, treatment recommendations for phyllodes tumors have involved surgical intervention with wide local excision and margins >1 cm, with the size of the tumor often determining whether a mastectomy is required. Malignant phyllodes tumors have a high potential for aggressive growth, local recurrence, and metastasis, commonly to the lungs, bones, and lymph nodes. Recent evidence suggests that margins <1 cm might be sufficient for adequate tumor excision, potentially allowing for breast-conserving surgery in certain cases. Thind et al. (2020) found no statistically significant difference in rates of local recurrence, metastasis, or survival between borderline and malignant phyllodes tumors resected with margins ≥1 cm or < 1 cm [5].

The role of adjuvant radiotherapy and chemotherapy in the treatment of phyllodes tumors is not clearly defined, largely due to a lack of prospective and randomized controlled studies on the subject [1]. Retrospective studies have shown that adjuvant radiotherapy can reduce local recurrence rates, especially in cases of high-risk malignant phyllodes tumors in patients who have undergone breast-conserving therapy; however, it has shown no effect on overall survival rates [6, 7]. The use of adjuvant chemotherapy is controversial and is primarily reserved for the management of metastatic disease, following NCCN guidelines for soft tissue sarcomas when employed [8, 9].

Despite advancements in understanding and treatment, the management of phyllodes tumors remains complex, requiring a tailored multidisciplinary approach based on individual tumor characteristics and patient factors.

## Conclusion

In conclusion, management of malignant phyllodes tumors remains complex due to their rare and variable nature. This case underscores the need for timely surgical intervention, given the tumor’s rapid growth and aggressive characteristics. Given the lack of an agreed upon consensus as to best management practices, tailoring treatment to individual tumor characteristics and employing a multidisciplinary approach are essential for optimizing patient outcomes. Continued research is crucial for refining management strategies and improving care for these challenging tumors.

## References

[1] Lissidini G, Mulè A, Santoro A, et al. Malignant phyllodes tumor of the breast: a systematic review. Pathologica 2022;114:111–20. 10.32074/1591-951X-754. 35414723 PMC9248247

[2] Zhou ZR, Wang CC, Yang ZZ, et al. Phyllodes tumors of the breast: diagnosis, treatment and prognostic factors related to recurrence. J Thorac Dis 2016;8:3361–8. 10.21037/jtd.2016.11.03. 28066617 PMC5179374

[3] Papas Y, Asmar AE, Ghandour F, et al. Malignant phyllodes tumors of the breast: a comprehensive literature review. Breast J 2020;26:240–4. 10.1111/tbj.13523. 31478587

[4] Tan BY, Acs G, Apple SK, et al. Phyllodes tumours of the breast: a consensus review. Histopathology 2016;68:5–21. 10.1111/his.12876. 26768026 PMC5027876

[5] Thind A, Patel B, Thind K, et al. Surgical margins for borderline and malignant phyllodes tumours. Ann R Coll Surg Engl 2020;102:165–73. 10.1308/rcsann.2019.0140. 31918563 PMC7027423

[6] Zeng S, Zhang X, Yang D, et al. Effects of adjuvant radiotherapy on borderline and malignant phyllodes tumors: a systematic review and meta-analysis. Mol Clin Oncol 2015;3:663–71. 10.3892/mco.2015.503. 26137284 PMC4471537

[7] Zhao W, Tian Q, Zhao A, et al. The role of adjuvant radiotherapy in patients with malignant phyllodes tumor of the breast: a propensity-score matching analysis. Breast Cancer 2021;28:110–8. 10.1007/s12282-020-01135-7. 32748225 PMC7796876

[8] Goodwin B, Oyinlola AF, Palhang M, et al. Metastatic and malignant phyllodes tumors of the breast: an update for current management. The American Surgeon™ 2023;89:6190–6. 10.1177/00031348231198114. 37611540

[9] Sars C, Sackey H, Frisell J, et al. Current clinical practice in the management of phyllodes tumors of the breast: an international cross-sectional study among surgeons and oncologists. Breast Cancer Res Treat 2023;199:293–304. 10.1007/s10549-023-06896-1. 36879102 PMC9988205

